# Alteration of Podocyte Protein Expression and Localization in the Early Stage of Various Hemodynamic Conditions

**DOI:** 10.3390/ijms14035998

**Published:** 2013-03-15

**Authors:** Kai Li, Juan Wang, Xiaohui Yin, Xiaoyue Zhai, Zilong Li

**Affiliations:** 1 Department of Surgical Oncology, First Affiliated Hospital of China Medical University, Shenyang 110001, China; E-Mail: cmu_likai@hotmail.com; 2 Department of Nephrology, First Affiliated Hospital of China Medical University, Shenyang 110001, China; E-Mails: cmuwoujken@sina.com (J.W.); yinxiaohuiv@126.com (X.Y.); 3 Department of Histology and Embryology, Institute of Pathology and Pathophysiology, China Medical University, Shenyang 110001, China; E-Mail: zhaixy@mail.cmu.edu.cn

**Keywords:** podocalyxin, nestin, acute hypertension, cardiac arrest, *in vivo* cryotechnique

## Abstract

Given that podocalyxin (PCX) and nestin play important roles in podocyte morphogenesis and the maintenance of structural integrity, we examined whether the expression and localization of these two podocyte proteins were influenced in the early stage of various hemodynamic conditions. Mice kidney tissues were prepared by *in vivo* cryotechnique (IVCT). The distribution of glomeruli and podocyte proteins was visualized with DAB staining, confocal laser scanning microscopy and immunoelectron microscopy. The mRNA levels were examined by real-time quantitative PCR. The results showed the following: Under the normal condition, PCX stained intensely along glomerular epithelial cells, whereas nestin was clearly staining in the endothelial cells and appeared only weakly in the podocytes. Under the acute hypertensive and cardiac arrest conditions, PCX and nestin staining was not clear, with a disarranged distribution, but the colocalization of PCX and nestin was apparent under this condition. In addition, under the acute hypertensive and cardiac arrest conditions, the mRNA levels of PCX and nestin were significantly decreased. Collectively, the abnormal redistribution and decreased mRNA expressions of PCX and nestin are important molecular events at the early stage of podocyte injury during hemodynamic disorders. IVCT may have more advantages for morphological analysis when researching renal diseases.

## 1. Introduction

The glomerular podocyte is a terminally differentiated cell that lines the outer aspect of the glomerular basement membrane (GBM). It forms the final barrier against protein loss, which explains why its dysfunction causes protein leakage into the urine, leading to proteinuria [1]. Podocytes are injured in many types of human and experimental glomerular diseases, including hypertensive renal disease [2–4]. The early events are characterized by molecular alterations of the slit diaphragm or by reorganization of the foot process structure with the fusion of filtration slits and apical displacement of the slit diaphragm [5–7].

Podocalyxin (PCX) is an extensively *O*-glycosylated and sialylated type I transmembrane protein that is normally expressed in the apical surface of kidney podocytes [8]. It is now thought to play an important role in podocyte morphogenesis and the maintenance of structural integrity through the negative charge of the PCX extracellular domain and by linking to the actin cytoskeleton to form junctional complexes between adjacent podocytes [9,10]. In animal models of glomerular malfunction attributed to abnormal PCX, the foot process architecture is disrupted, and slit diaphragms are displaced or completely replaced by leaky, discontinuous junctions. PCX-null mice have fewer major processes and lack foot processes and slit diaphragms [8].

Nestin is an intermediate filament protein originally described in neural stem cells and is recently thought to be expressed in differentiated podocytes of the adult kidney, which might be associated with the maintenance of the foot process structure [11,12]. Other study indicated that nestin is also expressed in vascular endothelial cells in the adult human pancreas [13]. Several studies on nestin expression in human renal diseases and animal models have been performed, but they produced conflicting results [11,14,15]. Based on the functions and possible linkage of PCX and nestin, we examined the changes in their expression and localization under various hemodynamic conditions, especially during the early stage, which is still poorly understood.

Hemodynamic factors, such as blood flow and pressure, are well-known to exert an important influence on the kidney structure and function, and these effects occur almost instantaneously [16]. However, the dynamic changes occurring in the podocyte component undoubtedly reflect various physiological and pathological statuses. Conventional tissue preparation methods, such as perfusion or immersion-fixation with chemical fixatives, must be performed when the heart has stopped and the circulation of blood has ceased, and the samples also require time to be prepared, which may affect the distribution of the molecular components and morphology. As a result, conventional tissue preparation methods cannot capture the split-second changes that occur in cells and tissues *in situ*[17–19].

For the past few decades, the *in vivo* cryotechnique (IVCT) has been used to immediately cryofix any target organ of living animals *in situ* without tissue resection, such as that needed with immersion-fixation or perfusion-fixation [18]. Remarkably, IVCT can immediately capture the biological constituents of cells and tissues, reflecting the actual biological function of the living state. In addition, IVCT has been used to clarify which serum proteins pass through the glomerular capillary loops under various hemodynamic conditions [20] and to describe the time-dependent double immunolocalization of intrinsic and extrinsic serum proteins at different time intervals using bovine serum albumin injection into cardiomyocytes [21]. The apparent advantages of IVCT make it a useful method for examining the expression and localization of podocyte proteins.

In the present study, we visualized the early distribution changes of podocyte proteins in living mouse glomeruli under various hemodynamic conditions by IVCT in combination with freeze-substitution and also described the quantitative analyses of these podocyte proteins. Our findings provide a new insight into the early molecular mechanisms of podocyte injury during hemodynamic disorders.

## 2. Results and Discussion

### 2.1. IVCT Exhibits a Clearer Morphological Alteration of the Glomeruli under Different Hemodynamic Conditions

**Figure 1:**
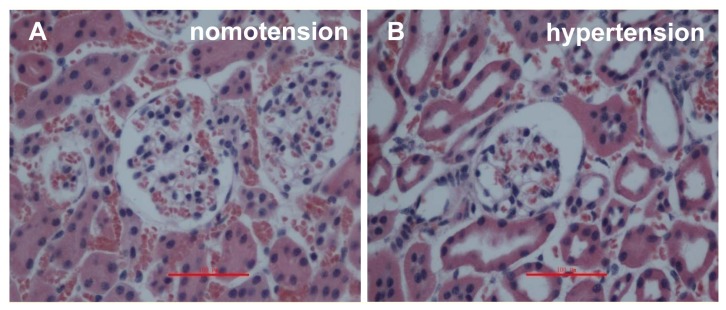

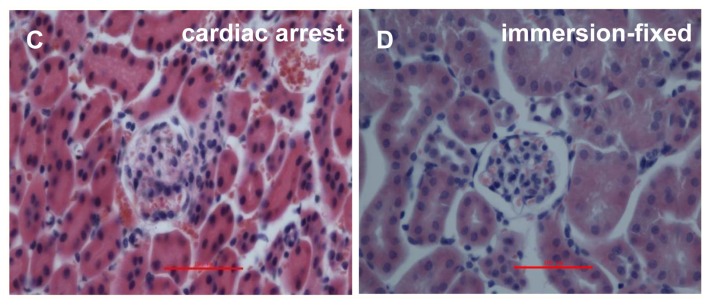
Light micrographs of mouse renal cortical tissues stained with hematoxylin-eosin, as prepared by the *in vivo* cryotechnique (IVCT) under normotensive (**A**), acute hypertensive (**B**), and cardiac arrest (**C**) conditions, as well as resection kidney tissue processed by the immersion-fixation method under normotensive condition (**D**). Under the acute hypertensive condition, the Bowman’s space and luminal spaces of the proximal and distal tubules were open widely in the renal cortices (**B**), and neither the Bowman’s spaces nor luminal spaces of the tubules were clearly open under the cardiac arrest condition (**C**). Scale bars = 100 μm.

To examine the native morphology in mouse kidneys from different groups, we stained the deparaffinized sections with hematoxylin-eosin (HE). For the sections obtained by IVCT, the capillary loops were smooth under a normotensive condition (Figure 1A); however, under the acute hypertensive condition, the Bowman’s space and luminal spaces of the proximal and distal tubules were open widely in the renal cortices (Figure 1B), and neither the Bowman’s spaces nor luminal spaces of the tubules were clearly open under the cardiac arrest condition (Figure 1C). In strong contrast to the IVCT images, the size of the glomeruli was obviously smaller, and the glomerular capillary loops (GCL) were partially shrunken in the immersion-fixed kidney tissues under normal condition (Figure 1D).

**Figure 2:**
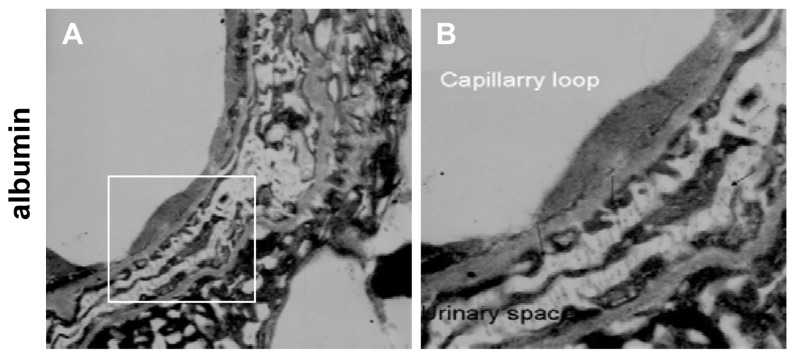

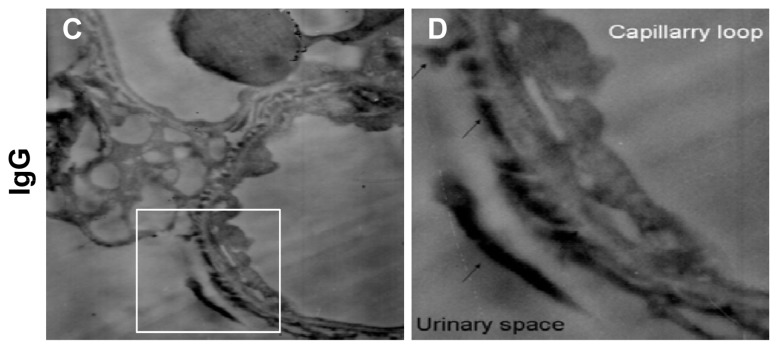
Immune electron micrographs of albumin and IgG in the glomerular filtration membrane under acute hypertensive conditions. Under the acute hypertensive condition, the distributions of albumin (**A**,**B**) and IgG (**C**,**D**) were changed. Both albumin and IgG were clearly immunolocalized along the apical surface of the podocytes and Bowman’s spaces (arrows). In addition, podocyte fusion and reduced microvilli could be observed. (Magnification, ×8000 for A and C, ×12000 for B and D).

In addition, to determine the instant ultrastructural changes of GBM under acute hypertensive conditions, the immunolocalization of serum proteins in GBM was observed by immunoelectron microscopy with the IVCT specimens. Under the acute hypertensive condition, both albumin (Figure 2A,B) and IgG (Figure 2C,D) were clearly immunolocalized along the apical surface of the podocytes and Bowman’s spaces (arrows). A high glomerular blood capillary pressure mechanically caused leakage of serum proteins, which passed through the slit diaphragm and GBM.

With IVCT, we obtained clear photographs of the glomeruli and GBM, which visibly showed the instant morphological alterations that occurred during the different hemodynamic conditions.

### 2.2. Instantaneous Changes in the Distribution and Expression of PCX and Nestin under Various Hemodynamic Conditions Are Examined by Immunohistochemistry and Immunofluorescence Analysis

**Figure 3:**
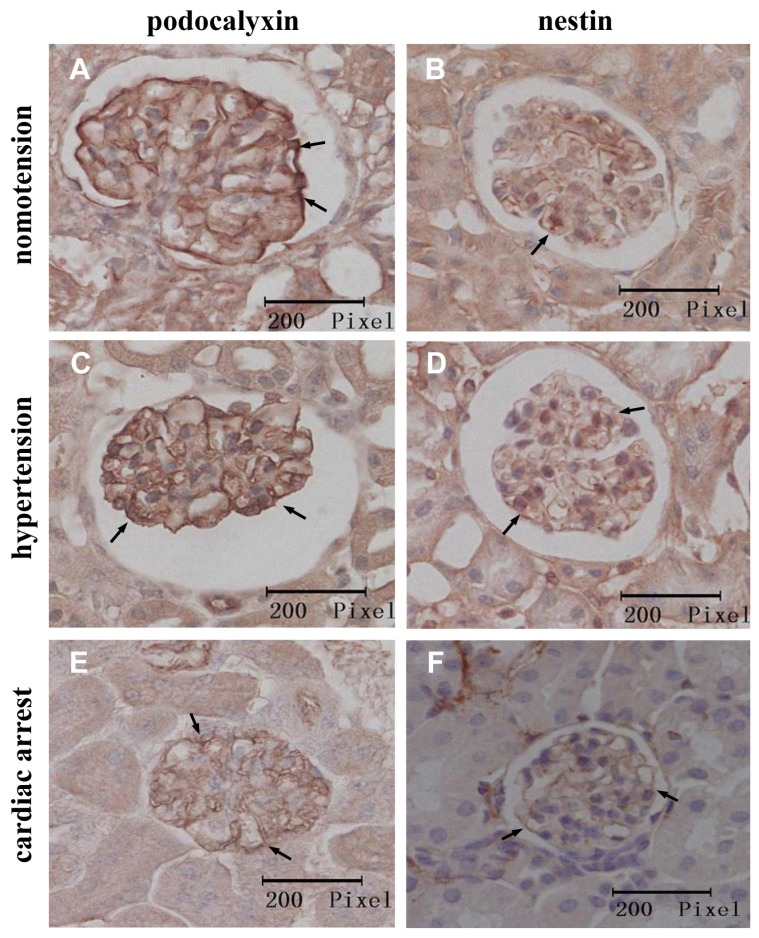

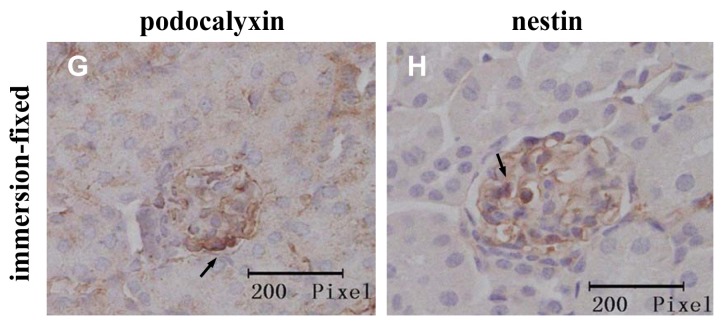
Immunohistochemical localization of the podocyte proteins in kidney tissues prepared with the IVCT and immersion-fixation methods under various hemodynamic conditions. The micrographs in the left column show the localization of podocalyxin (PCX) (arrows), and those in right column show nestin localization (arrows). The immunohistochemical localization of the two proteins prepared with IVCT are shown under the normotensive (**A**,**B**), acute hypertensive (**C**,**D**), and cardiac arrest (**E**,**F**) conditions. The tissues in G and H under normotensive condition were treated by the immersion-fixation method.

**Figure 4:**
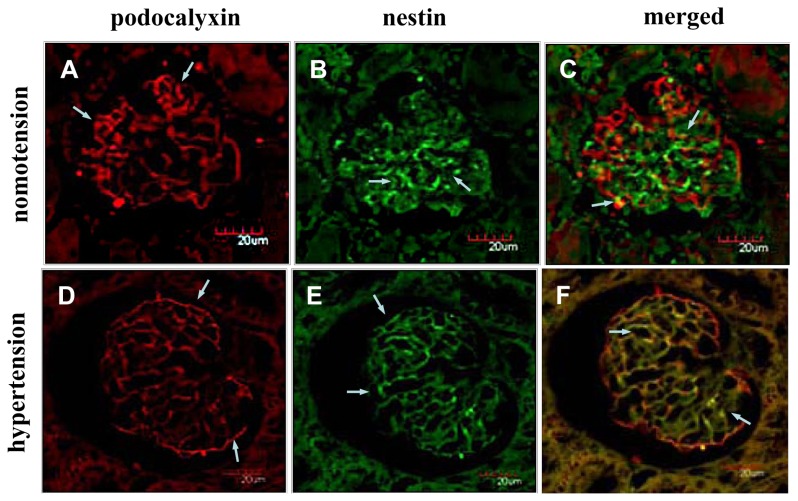

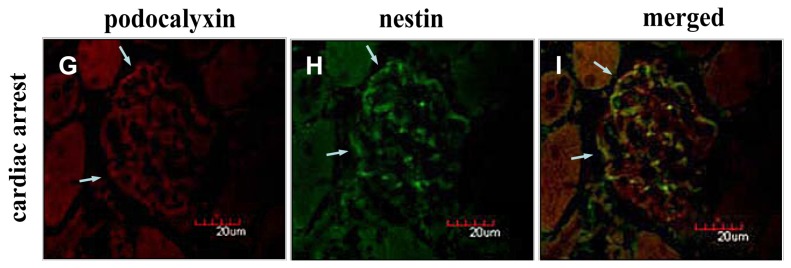
Immunofluorescence micrographs for PCX and nestin under various hemodynamic conditions. Confocal laser scanning micrographs show the double-fluorescence of PCX (red color) and nestin (green color). Under the normotensive condition, PCX showed an intense epithelial staining along the peripheral capillary loops of the glomeruli (**A**), and nestin showed an intense endothelial staining and a weaker appearance in the podocytes (**B**). Under the acute hypertensive condition, the immunoreactivity of PCX was decreased (**D**), and nestin immunolocalization was almost completely restricted to the podocytes (**E**). Under the cardiac arrest condition, the immunoreactivity of podocalyxin and nestin was reduced visibly compared with the normotensive condition, and nestin was restricted to the podocytes (**G**,**H**). The double-staining micrographs are shown in C (normotension), F (acute hypertension) and I (cardiac arrest).

Under the normotensive condition, PCX showed an intense epithelial staining along the peripheral capillary loops of the glomeruli, whereas nestin was mainly immunolocalized in the endothelial cells and showed slight immunostaining in the podocytes (Figure 3A,B). Under the acute hypertensive and cardiac arrest conditions, the PCX and nestin staining was weaker, occasionally showing a more granular appearance, as shown in Figure 3C–F. Additionally, the immunostained images from the immersion-fixation specimens showed smaller and shrunken glomeruli, in accordance with the HE staining results (Figure 3G,H). To clarify the relative immunolocalization of PCX and nestin in the glomeruli, we performed double immunofluorescence staining. The colocalization of PCX and nestin could be easily observed under the acute hypertensive condition compared with the normotensive condition (Figure 4A–F). Strikingly, in the cardiac arrest group, nestin was mainly deposited in the podocytes, and its deposition in the endothelia was visibly reduced; the expression of PCX was also decreased in the cardiac arrest condition (Figure 4G–I).

These results indicate that the acute hypertensive and cardiac arrest conditions decrease the expression of PCX and nestin and also influence the localization of these podocyte proteins.

### 2.3. Acute Hypertensive and Cardiac Arrest Conditions Decrease the mRNA Levels of PCX and Nestin in Kidney Tissues

**Figure 5:**
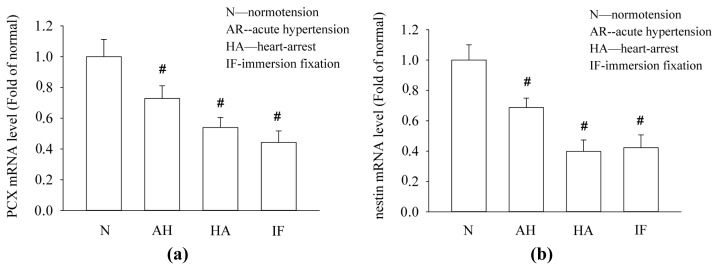
The effects of various hemodynamic conditions on the mRNA levels of PCX and nestin in kidney tissues. The mRNA levels of PCX and nestin in the kidney tissues under normotensive condition were prepared by the immersion-fixation method were also shown. The mRNA from kidney tissues was subjected to real-time quantitative PCR assays for PCX and nestin. The results are expressed as induction relative to the normotensive condition (mean ± SE), # *p* < 0.01.

To further evaluate the mRNA expression of PCX and nestin under different hemodynamic conditions, we examined the mRNA levels of these two podocyte proteins in kidney tissues. The results of the real-time quantitative PCR assays are shown in Figure 5. Obviously, the mRNA levels of PCX and nestin in the kidney tissues were consistently reduced under the conditions of hemodynamic change compared with the normotensive condition. Additionally, the mRNA levels of PCX and nestin in the kidney tissues under normotensive condition were prepared by the immersion-fixation method were also presented.

The reduction in the PCX and nestin expression in the kidney tissues is consistent with the immunostaining results and could be attributed to the alteration of the renal hemodynamic conditions and different tissue preparation process.

### 2.4. Discussion

In this study, we described, for the first time, the alteration of podocyte proteins caused by various hemodynamic conditions at the early stage of the process, which was demonstrated by IVCT combined with immunohistochemistry and real-time quantitative PCR assays. We found that the expression of PCX and nestin decreased and that the localization of the two proteins was abnormally redistributed under the acute hypertensive and cardiac arrest conditions compared with the normotensive condition.

To the best of our knowledge, hemodynamic factors, such as blood flow and pressure, influence the structure and function of the kidney. Because these hemodynamic factors can change instantaneously [18], we need a technology that can instantly capture the condition of the tissues and cells *in situ* to study these changes and reflect their actual pathological and physiological statuses. With traditional tissue preparation techniques, such as perfusion-fixation or immersion-fixation, many components are washed away or displaced, which can lead to inaccurate results [18–20]. However, IVCT provides an effective method that preserves all biological components. This technique overcomes the technical problems of conventional fixation processes by cryofixing the target organs *in situ* under a living status, which may clarify the native morphological features of the kidney tissues [18,20]. In this study, IVCT preserved more podocyte proteins than in the resected kidney tissues; at the same time, IVCT captured more molecular events instantly *in situ*, which was much closer to the living state. The immunoelectron microscopy of the serum protein immunolocalization in GBM clearly demonstrated that the high pressure of the glomerular blood capillaries impaired the size-selective barrier function of the slit diaphragm and GBM. The results were similar to those from a previous report [22].

Under the normal hemodynamic condition, PCX showed an intense epithelial staining along the peripheral capillary loops of the glomeruli, similar to previous reports [23–25]. In contrast, under the acute hypertensive condition, PCX was mainly deposited along the outer side of the glomerular capillaries, and the capillaries were crimped compared with the normal glomeruli. The decrease and redistribution of PCX might suggest an uncoupling of PCX from the actin cytoskeleton as a result of a rearrangement of the cytoskeleton. This phenomenon has been described in rat models of long-term high glucose exposure, in which the complex linking of PCX to the actin cytoskeleton is disrupted [26,27].

With IVCT, nestin showed an interesting phenomenon. There was intense endothelial staining and a weaker appearance in the podocytes, which differed from previous reports using conventional preparation methods, in which nestin was expressed in terminally differentiated podocytes in mature kidneys [11,28,29]. Additionally, the immunolocalization of nestin in glomerular endothelial cells was obviously decreased under the acute hypertensive and cardiac arrest conditions. It is important to emphasize that the expression of nestin is not limited to podocytes in mature kidneys. It is also expressed in endothelial cells, which can explain why epithelial injury also involves proteinuria. Ritz *et al.* indicated that renal damage secondary to hypertension may be mediated by injury to endothelial cells [30], and in subsequent studies, endothelial cells were reported to play a vital role in hypertensive renal damage [31]. Deen also demonstrated that proteinuria can occur with endothelial disruption alone [32]. Preeclampsia, which is characterized by new-onset hypertension and proteinuria, in association with a characteristic glomerular lesion, endotheliosis, further revealed the subtle relationship among hypertension, endothelial cells and proteinuria [33,34]. It is worthwhile to highlight that nestin may play a vital role in the occurrence and development of proteinuria, being involved not only in podocyte dysfunction but also in endothelial cell lesions.

In this study, the mRNA levels of nestin were reduced under the acute hypertensive and cardiac arrest conditions, which is similar to the PCX expression. In previous studies, nestin expression in kidney tissues under pathological conditions was controversial. In puromycin aminonucleoside nephrosis of rats, the expression of nestin increased [15]. Perry *et al.* reported that many diseases (e.g., minimal lesion, FSGS, IgA nephropathy and lupus nephritis) showed nestin expression in the kidney tissues at similar or increased levels compared with the normal state [14]. Nevertheless, in Alport syndrome and thin GBM disease, the expression of nestin was reduced. Su *et al.* reported a down-regulation of nestin expression in FSGS, MN and IgA nephropathy with proteinuria, and the glomerular nestin expression levels correlated inversely with the 24-hour urine protein results [11]. Based on the advantages of IVCT for preserving biological components and capturing instantaneous changes in tissues, our observations provide an early picture of nestin localization and expression during the acute hypertensive and cardiac arrest conditions, but subsequent changes that occur must be proven with further research.

In addition, the ectopic immunolocalization of PCX and nestin in the glomeruli during acute hypertension might be attributed to the acute increased pressure in the glomeruli that occurs with the ligation of distal renal arteries branching off the aorta [16,20]. In our study, we observed the displacement of podocyte proteins during an acute hypertensive state because the higher pressures in the glomerular capillaries mechanically changed the structure of the podocytes and impaired the glomerular filtration barrier.

## 3. Experimental Section

### 3.1. Antibodies

The rabbit polyclonal antibody against PCX, mouse monoclonal antibody against nestin, biotinylated sheep anti-rabbit IgG and biotinylated rabbit anti-mouse IgG were purchased from Sigma (St. Louis, MO, USA). The Alexa Fluor 488-conjugated donkey anti-rabbit IgG and Alexa Fluor 594-conjugated donkey anti-mouse IgG antibodies were obtained from Abcam (Cambridge, MA, USA).

### 3.2. Animals

Adult female C57BL/6J mice, weighing 25 to 30 g, were used. The animal experimental procedures were approved by the Animal Experimental Committee of China Medical University.

### 3.3. Preparation of Kidney Tissues

#### 3.3.1. IVCT for Mouse Kidneys, Freeze-Substitution Fixation and Paraffin-Embedding

We divided 15 mice into three groups of five mice each: a normotensive group, an acute hypertensive group, and a cardiac arrest group. We anesthetized the mice via the intraperitoneal injection of sodium pentobarbital (50 mg/kg body weight). In the normotensive group, we removed the left kidneys during normal blood circulation. In the acute hypertensive group, we prepared an animal model with acute renal hypertension by the ligation of the abdominal aorta just below the branching renal arteries for 10 min [20] before removing the left kidneys. In the cardiac arrest group, the blood flow into the kidneys was ceased by stopping their beating hearts with an intra-abdominal injection of excessive amounts of the anesthetic. After the injection, IVCT was performed at a time interval of 5 min. These mice were automatically monitored using an electrocardiogram (ECG) apparatus when the IVCT was performed [19], and the left kidneys were then removed.

After obtaining the renal tissues, we performed IVCT according to our previous report [16,20]. Briefly, a cryoknife precooled in liquid nitrogen (−196 °C) was positioned over the left kidney of an anesthetized mouse. The kidney was immediately cut with the cryoknife, and liquid isopentane-propane cryogen (−193 °C) was simultaneously poured over the kidney. The frozen kidney tissues *in vivo* were carefully trimmed with a dental electrical unit in liquid nitrogen. Some pieces of the trimmed frozen kidney tissue were processed for the next freeze-substitution step as described below, and the remainder was preserved in liquid nitrogen for western blotting.

The pieces were freeze-substituted in pure acetone containing 2% paraformaldehyde (PFA) at −80 °C in dry ice-acetone for 48 h and then gradually warmed to room temperature. They were washed in pure acetone twice, transferred into xylene and then embedded in paraffin wax.

#### 3.3.2. Immerse-Fixation of Resected Kidney Tissues

As a control group, we resected the left kidneys of another five mice under anesthesia, and a portion of the kidney tissues were quickly plunged into 4% PFA. After 24 h at room temperature, they were gradually dehydrated in alcohol and transferred into xylene. Finally, they were embedded in paraffin wax, and the remainder of the tissue was preserved in −80 °C for the biochemical examination.

### 3.4. Immunostaining on Deparaffinized Sections

The paraffin-embedded tissues were cut at a 5 μm thickness and deparaffinized with xylene and a graded series of ethanol. We stained some sections in common hematoxylin and eosin (HE) to obtain the morphological findings. Other sections were incubated with 1% hydrogen peroxidase (H_2_O_2_) to block the non-specific reactivity of endogenous peroxidase. After washing in phosphate buffered saline (PBS), the sections were repaired with sodium citrate buffer liquid at high pressure and incubated with PBS containing 5% normal bovine serum (Boster, Wuhan, China) for 1 h at 37 °C, followed by incubation in the primary antibodies at 4 °C overnight and corresponding secondary antibodies at 37 °C for 1 h. Then, the sections were incubated with a horseradish peroxidase (HRP)-conjugated avidin-biotin complex (ABC) for 20 min, and the staining was visualized with metal-enhanced 3,3′-diaminobenzidine (DAB) (Boster, Wuhan, China) for 5 min (ABC-DAB method). The sections were also stained with hematoxylin for 1 min, and the stained sections were dehydrated with a graded series of ethanol and xylene. Finally, they were sealed with peucine and photographed under a light microscope or a confocal laser scanning microscope (FV10-ASW2.1Viewer).

### 3.5. Immunoelectron Microscopy

To examine the glomerular leakage of the serum proteins, albumin and IgG, under acute hypertensive conditions, we stained some sections after DAB staining with 1% osmium tetroxide acid for 20 min. The sections were then dehydrated with a graded series of ethanol and acetone and finally inversion embedded with Epon 812 polymerized at 60 °C for 48 h. The sections were cut at a 70 nm thickness and stained with uranyl acetate, and then ultrastructural images were taken with a transmission electron microscope.

### 3.6. Real-Time Quantitative PCR

Total RNA was extracted using the TRIzol reagent from the renal cortex according to the manufacturer’s procedure. The RNA concentration and quality were assessed by spectrophotometry at 260 and 280 nm, cDNA was generated using the Takara cDNA synthesis kit according to the manufacturer’s protocol, and the RNA were reverse-transcribed using the follow procedure: 37 °C for 5 min, followed by 85 °C for 5 s. The specific primers are as follows: PCX (forward 5′-CTTGAGACACAGACACAGAG, reverse 5′-CCGTATGCCGC--ACTTATC); nestin (forward 5′-AAGCAGGGTCTACAGAGTCAGATCG, reverse 5′-GCTGTCACAGGAGTCTCAAGGGTAT); β-actin (forward 5′-TGGCACCCAG-CACAATGAA, reverse 5′-CTAAGTCATAGTCCGCCTAGAAGCA). The reaction system included the following in a total volume of 20 μL: the upstream and downstream primers of podocalyxin, nestin and β-actin each 0.5 μL, SYBR Premix Ex Tap 10.5 μL, cDNA 2.0 μL, and deionized H_2_O 6.5 μL. The PCR cycling parameters were as follows: denaturing at 95 °C for 30 s once; 95 °C for 5 s and 60 °C for 34 s, repeated for 40 cycles; and 95 °C for 15 s, 60 °C for 1 min, and 95 °C for 15 s on the last cycle. ΔΔCt = (Ct_target gene_ − Ct_internal reference_) − (Ct_target genes of control group_ − Ct_internal reference in the control group_), mRNA relative expression level = 2^−ΔΔCt^.

### 3.7. Statistical Analysis

The values are expressed as the mean ± SE. For multiple comparisons with a single control, one-way analysis of variance (ANOVA) followed by Dunnett’s test was employed. The analyses were conducted using SigmaStat statistical software (Jandel Scientific, San Rafael, CA, USA). *p <* 0.05 was considered to be a statistically significant difference.

## 4. Conclusions

Collectively, using IVCT, we visualized the initial alterations of PCX and nestin expression and localization under various hemodynamic conditions and revealed an important molecular mechanism for podocyte injury during hemodynamic disorders. In addition, IVCT has multiple advantages, not only for morphological analyses but also for the biochemical examination, for the continued research of renal diseases.
